# Case Report: Oral glutamatergic augmentation for trauma-related disorders with fluoxetine-/bupropion-potentiated dextromethorphan ± piracetam: a four-patient case series

**DOI:** 10.3389/fpsyt.2026.1752101

**Published:** 2026-05-12

**Authors:** Ngo Cheung

**Affiliations:** Cheung Ngo Medical Limited, Hong Kong, Hong Kong SAR, China

**Keywords:** AMPA, glutamatergic, NMDA, PTSD, trauma

## Abstract

Traditional monoaminergic medications often offer limited relief for the physical and cognitive symptoms of post-traumatic stress disorder (PTSD) and complex PTSD. Growing data now point to fast-acting, glutamate-based treatments that boost synaptic plasticity and interrupt fear-conditioned neural circuits. We report four sequential cases of hard-to-treat trauma-spectrum disorders—somatic PTSD, acute bereavement-related PTSD, trauma-linked adolescent depression, and complex PTSD complicated by bipolar II disorder, ADHD, and borderline features—that showed clinically meaningful symptom improvement, typically within days to weeks, with an inexpensive, fully oral protocol centred on dextromethorphan (DXM) potentiated by fluoxetine, with optional add-on piracetam and/or bupropion. All four patients showed notable reductions in intrusive memories, rumination, somatic pain, and functional disability; no episodes of dissociation, hypertension, or mania were clinically documented during follow-up, although structured screening for hypomania/mania and serotonergic toxicity was not performed. These findings are strictly hypothesis-generating and broaden the ketamine/Auvelity framework to trauma-spectrum presentations. They suggest that further controlled investigation into oral NMDA–AMPA modulators may be warranted for trauma-related conditions.

## Introduction

Growing evidence frames trauma-related disorders as problems of maladaptive synaptic wiring: fear memories are over-encoded, extinction pathways are weak, and prefrontal-limbic plasticity is depleted (1, 2). Intravenous ketamine and multi-dose esketamine can lift PTSD symptoms within hours by briefly blocking NMDA receptors, provoking an AMPA-mediated glutamate surge, and activating the BDNF–mTOR pathway that drives new synapse formation (3, 4). Approval of the oral dextromethorphan–bupropion combination Auvelity^®^ for major depression shows that this mechanism can be replicated with a daily pill, yet the drug is hard to obtain in many places and has not been tested in “pure” trauma disorders (5).

Here we describe four cases in which a practical, entirely oral glutamatergic strategy—dextromethorphan boosted by fluoxetine to extend NMDA antagonism, with optional low-dose piracetam for AMPA facilitation and/or bupropion for extra CYP2D6 inhibition and catecholamine support—was temporally associated with rapid symptom improvement hypothesised to involve mechanisms similar to those of ketamine. Symptom reduction was seen across the trauma spectrum, from somatic amplification and acute bereavement-related PTSD to adolescent rumination and complex PTSD with multiple comorbidities. No serious adverse events were clinically documented during follow-up, and functional gains included return to work and social engagement in patients who had previously failed standard treatments.

## Methods

This report reviews four back-to-back cases of trauma-related illness managed with an oral glutamatergic add-on protocol at a private outpatient clinic (Tsim Sha Tsui H Zentre Clinic, Cheung Ngo Medical) between February 2024 and November 2025. Every patient was personally assessed and treated by the author.

As this was a retrospective review of routine clinical care, formal IRB/ethics committee approval was not required per Hong Kong private-clinic policy; written informed consent for publication was obtained from all four patients (and from parents plus written assent from the adolescent). All identifying details have been removed or generalised to minimise re-identification risk.

Safety was monitored via clinical interview, spontaneous patient report of adverse events, and vital signs (including blood pressure) at every follow-up visit (every 2–8 weeks).

Four consecutive eligible trauma-spectrum patients who provided publication consent were included.

The core schedule used dextromethorphan hydrobromide 15 mg tablets (usual total dose 30–90 mg/day in divided doses) whose exposure was prolonged by a potent CYP2D6 blocker—either fluoxetine 10–20 mg/day or bupropion XL 150 mg/day. Piracetam 600–1–200 mg/day could be added as an AMPA-positive allosteric modulator. Doses were titrated case-by-case for benefit and tolerability. Pre-existing psychotropics were left in place unless patients chose otherwise. Follow-up visits occurred every 2–8 weeks, combining PHQ-9 and GAD-7 self-ratings with an in-depth clinical interview; formal OCD or PTSD instruments were not used in routine care.

## Case 1: symptom improvement in a patient with somatic-predominant post-traumatic stress features with fluoxetine-potentiated dextromethorphan

### Case presentation

A 26-year-old Chinese male (hereafter Mr X) first attended the outpatient clinic in early February 2024, approximately two years after an undisclosed traumatic event that had precipitated persistent post-traumatic stress and diffuse bodily pain. He was unemployed, sleeping poorly, and experiencing daily neck-to-body aches so intense that he “wanted to pass out.” Panic attacks occurred about once a day. On examination he appeared anxious, clasped the back of his neck, and displayed mild whole-body tremor. No neurological deficit was found. Initial management consisted of low-dose fluoxetine 10 mg daily together with alprazolam, risperidone 0.5 mg, lemborexant for sleep, and short courses of benzodiazepine hypnotics. However, he defaulted the follow-up as he did not believe he had psychiatric issues.

The clinical picture fulfilled ICD-11 criteria for Post-Traumatic Stress Disorder. The patient described daily, vivid flashbacks of a single, undisclosed traumatic event and displayed strong, often unconscious avoidance, exemplified by two years of unemployment and marked social withdrawal. A pervasive sense of current threat was evident in pronounced hypervigilance, exaggerated startle responses, recurrent panic attacks, and a persistent state of somatic hyper-arousal that manifested as diffuse pain, neck stiffness, and tremor. Although these bodily complaints dominated the presentation, they were conceptualised as an amplified expression of PTSD rather than a separate disorder—what some authors term “somatic PTSD.” The illness had persisted well beyond one month and caused substantial functional impairment; no features of Complex PTSD were present. DSM-5-TR criteria were likewise met. Panic Disorder was considered but not formally assessed.

### The use of fluoxetine + DXM regimen

On 24 June 2024, he returned and requested medication as advised by his counsellor. The treatment strategy was revised. Dextromethorphan (DXM) 45 mg in total daily was introduced and fluoxetine was simultaneously increased to 20 mg daily. The remainder of his psychotropic background—risperidone 0.5 mg, low-dose quetiapine, pregabalin 25 mg nocte, and the same hypnotic support—was left unchanged. Because fluoxetine is a potent cytochrome P-450 2D6 inhibitor, this adjustment was expected to raise and prolong circulating DXM levels.

### Treatment course and functional outcomes

A notable clinical change followed. At the next review (19 August 2024) Mr X reported that the all-day lancinating pain had dwindled to a dull ache and now surfaced only sporadically. Flashbacks had fallen from daily to roughly once a week; sleep consolidated to 6–7 hours with far fewer nocturnal awakenings. Standardised self-report scores mirrored the subjective change (PHQ-9 = 6, GAD-7 = 5). By mid-September, pain was “minimal,” flashbacks had ceased, and hypervigilance had given way to relaxed evening gaming sessions. Objective scores plateaued (PHQ-9 = 6, GAD-7 = 2), suggesting residual mood-somatic discord but continued momentum toward recovery.

Functional gains soon followed. In October Mr X accepted a junior accounting post, sustaining full-time hours without absenteeism—an important milestone after two years of occupational inactivity. Concurrent questionnaires continued to drift downward; by November PHQ-9 was 3 and GAD-7 was 2, and the patient described only mild morning sleepiness as a remaining nuisance.

The improvement proved durable. Between February and August 2025 mood and anxiety ratings stabilised at the floor (PHQ-9 = 0–1; GAD-7 = 0–1). Mr X achieved marked and sustained improvement in somatic pain, flashbacks, and function. Somatic complaints vanished, including the incapacitating neck and body pain that had dominated the initial presentation. Mr X maintained his accounting position, began preparations for the CPA examination, and re-engaged in leisure pursuits such as vintage-car restoration. No clinically documented episodes of dissociation, hypertension, serotonergic toxicity, or mania were recorded throughout 14 months of follow-up. Safety monitoring consisted of clinical interview, spontaneous patient reports, and vital signs including blood pressure; structured scales for hypomania or serotonin syndrome were not used.

### Mechanistic discussion on the treatment of PTSD somatisation with a glutamatergic strategy

It is hypothesised that the observed temporal association between treatment initiation and symptom reduction in this patient may relate to trauma-induced neuro-interoceptive dysregulation. Chronic threat exposure sensitises the hypothalamic–pituitary–adrenal axis and biases salience networks toward the body, a process that amplifies innocuous visceral or musculoskeletal signals into distressing pain and fatigue (6, 7). Functional neuroimaging reveals concurrent hyper-responsivity of the anterior cingulate and insula—centres that amalgamate emotion with interoception—in both post-traumatic stress disorder (PTSD) and somatic symptom disorder (8). Once this circuitry is in place, it creates a positive feedback loop: physical discomfort leads to hypervigilance and re-experiencing, which then makes somatosensory amplification even stronger (7, 9).

Emerging work on ketamine suggests that quickly boosting glutamate transmission and downstream BDNF–mTOR signalling might rebuild atrophied synapses throughout the amygdala-hippocampus-mPFC circuit in chronic PTSD (3, 4). Dextromethorphan (DXM) functions as a low-affinity, rapidly dissociating NMDA antagonist and simultaneously activates σ-1 receptors that promote BDNF release and neuroplasticity (5). When DXM is co-formulated with bupropion, two additional advantages appear. First, bupropion’s strong CYP2D6 inhibition slows DXM metabolism, maintaining plasma levels long enough to open a sustained but tolerable “plasticity window.” Second, bupropion’s dopaminergic–noradrenergic re-uptake blockade can counter the hypo-reward and fatigue that often perpetuate bodily hyper-vigilance in traumatised patients. In principle, therefore, a bupropion + DXM tablet may deliver a miniature, orally bioavailable approximation of ketamine’s hypothesised circuit-repairing signal—one that could be taken daily and paired with exposure-based therapies.

For patients whose PTSD is dominated by medically unexplained pain, dizziness, or gastrointestinal distress, this mechanism is especially attractive. Somatic amplification has been linked to hypersynchronous firing between limbic salience hubs and interoceptive cortex; ketamine normalises that coupling within 24 h (1). Pre-clinical data suggest σ-1 modulation further stabilises autonomic outflow and visceral sensory gain, while dopaminergic tone restores top-down gating of innocuous body signals (10). By extending DXM exposure, bupropion may therefore blunt both the emotional flashback and its physical echo, a hypothesis consistent with the rapid fade-out of diffuse pain and tremor observed in our case. Although formal PTSD trials have yet to be conducted, the combination’s pharmacology aligns with the glutamatergic plasticity model that now underpins ketamine’s success. A next step is a small, mechanistic study measuring changes in insula–amygdala connectivity and daily somatic-symptom diaries after six weeks of bupropion + DXM, ideally in conjunction with brief exposure therapy. If the neural and bodily signals quiet in tandem, this inexpensive oral regimen could become a practical bridge between sophisticated psychoplastogens and routine outpatient PTSD care.

## Case 2: symptom improvement in acute bereavement-related trauma symptoms with a neuroplasticity-focused pharmacological approach

A 28-year-old woman who worked as a public-sector nurse presented to our outpatient clinic in April 2025 with profound dysphoria, severe anxiety, and insomnia precipitated by the sudden suicide of her mother two weeks earlier. She had been followed in the clinic for bipolar affective disorder since mid-2024 and had experienced fluctuating depressive episodes despite trials of escitalopram, venlafaxine, lamotrigine, aripiprazole, quetiapine, and multiple hypnotics. Prior to the bereavement her symptoms were under partial control, with a PHQ-9 score of 9 and adequate occupational functioning in a non-clinical post.

### Acute post-traumatic decline

The death of her mother triggered an acute stress reaction characterised by persistent intrusive images of the jump, exaggerated startle, survivor guilt, and nightly nightmares. Within one month her PHQ-9 rose to 27 and GAD-7 to 21; she described pervasive hopelessness, emotional numbing, and passive suicidal ideation but denied active intent. Short courses of fluoxetine and risperidone were added without meaningful change. By June 2025 she was sleeping up to 14 hours yet felt “drained,” complained of cognitive fog, and had begun to avoid previously enjoyed volunteer work. Dextromethorphan 30 mg daily was introduced at that visit for its rapid-acting antidepressant potential (considering she had been taking fluoxetine, which is a potent CYP2D6 inhibitor). Although she reported a brief lift in energy, adherence was poor and her mood relapsed; in late July she again endorsed severe depressive and anxiety symptoms related to continuing workplace harassment and intrusive trauma memories.

At intake the patient met ICD-11 criteria for PTSD precipitated by the sudden suicide of her mother, an event that clearly satisfies Criterion A. She reported relentless intrusive images of the scene, nightly trauma-related nightmares, emotional numbing, and withdrawal from previously valued activities such as volunteer work. A heightened sense of threat was reflected in survivor guilt, severe anxiety, hypervigilance, and an exaggerated startle reflex, all accompanied by hypersomnia and a marked decline in occupational functioning. Symptoms had been present for more than one month. The episode occurred on a background of Bipolar II Disorder, which appeared secondarily worsened but did not account for the clinical picture. Early pharmacological and psychosocial intervention was associated with rapid symptom improvement and functional recovery. DSM-5-TR criteria for PTSD were fully satisfied, and no additional features suggested Complex PTSD.

### Multimodal neuroplasticity-focused strategy

Because of the partial and transient response, a multimodal strategy was started in early September 2025. Piracetam 600 mg twice daily was prescribed on 4 September to augment neuroplasticity, and dextromethorphan was reinstated at 30 mg daily (later increased to 60 mg). Nine days later bupropion XL 150 mg daily was initiated both for bipolar-depressive symptoms and to raise systemic dextromethorphan exposure through CYP2D6 inhibition. No agitation, sleep disruption, or other clinically apparent adverse events were documented during this period. Within three weeks she reported markedly brighter affect, spontaneous engagement in social activities, and resolution of hypersomnia. Nightmares diminished from nightly to once weekly, and flashbacks became “distant and blurry.” By 10 November 2025 her PHQ-9 had fallen to 4 and GAD-7 to 0; she had accepted a new nursing position and was planning postgraduate study. Mild hand tremor responded to propranolol 10 mg as needed, and no manic switch was clinically documented during follow-up; structured screening for hypomania was not performed.

### Current status and safety monitoring

At the most recent review she continued the triple regimen of bupropion, dextromethorphan 60 mg/day, and piracetam 1–200 mg/day alongside maintenance lamotrigine 100 mg and low-dose aripiprazole. Depressive symptoms remain at minimal levels (PHQ-9 = 4), though occasional intrusive thoughts of her mother persist, consistent with evolving post-traumatic stress disorder. She has resumed full-time work, maintains regular sleep without hypnotics, and demonstrates good insight and adherence. No clinically significant hypertension, psychosis, or hepatotoxicity was documented over three months of combination therapy; however, monitoring relied on clinical interview, spontaneous patient reports, and vital signs rather than structured scales for mania/hypomania, serotonergic toxicity, or systematic hepatic function panels.

### The pharmacologic “plasticity window”

These observations raise the hypothesis that early augmentation of trauma-focused psychotherapy with a bupropion + dextromethorphan + piracetam regimen might have engaged some of the plasticity mechanisms now being explored with ketamine and psychedelics (12). Dextromethorphan’s low-affinity NMDA antagonism is similar to how ketamine can “lift the brake” on mTOR/BDNF cascades, which quickly reverses stress-induced synaptic loss in limbic–frontal circuits (1, 4). Structural crystallography has demonstrated that piracetam binds at the dimer interface of AMPA receptor subtypes GluA2 and GluA3, consistent with weak positive allosteric modulation (11), though its clinical significance for plasticity in humans remains to be firmly established. Bupropion’s catecholaminergic boost likely sustains the metabolic and motivational milieu required for synaptogenesis, much as increased arousal during exposure sessions predicts greater prefrontal–hippocampal integration (10). In effect, the triple combination may, in theory, approximate aspects of the transient neuroplasticity enhancement attributed to ketamine—buying precious days in which psychotherapy can reconsolidate traumatic memories into adaptive semantic networks.

This framework fits with evidence that combining memory reactivation with plasticity-enhancing agents lowers amygdala hyper-reactivity and boosts top-down control, mechanisms that have been proposed to facilitate adaptive reconsolidation of traumatic memories and support symptom resolution (3, 13). The regimen may have contributed to the rapid symptom improvement and functional recovery observed in this case. The rapid fall in PHQ-9/GAD-7 scores, the reduction in hypervigilance, and the patient’s restored occupational functioning are consistent with this neurobiological hypothesis. Although controlled trials remain essential, our case supports the hypothesis that inexpensive, repurposed agents can be combined rationally to target neuroplasticity-related mechanisms and may contribute to symptom reduction in trauma-related presentations.

## Case 3: reduction in rumination and associated symptoms in an adolescent with trauma-associated depression

### Case presentation

A 13-year-old secondary-school girl was referred to our clinic in early November 2025 after her class teacher observed that she had become tearful and distracted during lessons. One month earlier the same teacher had contacted the patient’s mother to report escalating emotional outbursts in class. At consultation the mother was visibly distressed and worried that her own anxiety might be fuelling her daughter’s decline.

### Trauma back in 2 years ago

The adolescent traced the onset of her low mood to an incident in Primary 6 when a close friend divulged personal confidences, followed her home and subjected her to repeated teasing. In the weeks that followed she engaged in superficial self-injury on campus and developed a persistent conviction that the humiliation could recur at any time. Although she progressed academically and maintained several friendships, the memory of that betrayal left what she described as a “shadow.” She now worried constantly that incomplete homework or imperfect test scores would bring shame on her family, keep her awake at night and, most upsettingly, make her mother cry. She admitted to lying awake rehearsing these fears until exhaustion forced sleep and reported little relief from daytime distractions such as choir practice, an activity she ordinarily enjoyed.

At intake she scored 23 on the PHQ-9 and 21 on the GAD-7, indicating severe depressive and anxious symptomatology. Physical examination and routine laboratory results were unremarkable. The clinical impression was a trauma-coloured major depressive episode characterised by intrusive ruminations and guilt-laden catastrophising.

This adolescent fulfilled ICD-11 criteria for a Major Depressive Episode with prominent trauma-related features secondary to repeated childhood relational trauma. Although full PTSD criteria were not reached, partial features were present: intrusive, shame-laden recollections of the interpersonal trauma, covert avoidance of social risk through perfectionism, and a persistent sense of threat characterised by nocturnal rumination and hypervigilance to social cues. Depression was severe, with PHQ-9 and GAD-7 scores of 23 and 21, respectively, and included obsessive–compulsive–like perseverative thinking. DSM-5-TR criteria for Major Depressive Disorder with anxious distress were met; elements reminiscent of Persistent Complex Bereavement Disorder were considered but the inciting trauma was interpersonal, not bereavement. No disturbances in self-organisation pointed toward Complex PTSD.

### The regimen to interrupt ruminations quickly

Because the emotional loops appeared resistant to reassurance alone, we initiated a pharmacological programme aimed at interrupting rumination quickly while minimising sedation. The backbone consisted of dextromethorphan 30 mg nightly, piracetam 600 mg nightly in combination with fluoxetine 10 mg each morning. Low-dose risperidone 0.25 mg daily was added to dampen obsessive ideation.

Twelve days later the patient returned with a noticeably lighter affect. She reported feeling “less stuck” on thoughts of the primary-school event and no longer combed through every classroom interaction for signs of betrayal. Homework was still completed diligently, yet the nightly compulsion to perfect every detail had eased, allowing her to claim personal “me-time” before bed and to fall asleep without prolonged mental replay. Choir rehearsals were once again experienced as pleasurable rather than obligatory. Her PHQ-9 had fallen to 15 and the GAD-7 to 13, both in the moderate range. She attributed the change to “the medicine making my brain quieter,” and neither she nor her mother observed adverse effects such as dizziness, dissociation or gastrointestinal upset.

Given the favourable response we continued her regimen. No additional hypnotic or anxiolytic agents were deemed necessary. A support letter was provided to the school outlining accommodations, though the patient reported she already felt markedly less fearful about attending classes.

### Integrating the patient’s course with the broader science

By the November follow-up, the patient’s mood had improved faster than expected for a first-episode adolescent depression. The bullying she experienced three years earlier had left a prolonged “shadow” that was not simply an adjustment to her current school stressors. Trauma-linked depression, unlike purely situational depression, is interwoven with post-traumatic stress disorder (PTSD) physiology: intrusive recollections of the event, hypervigilance, and concrete, event-focused rumination (14–16). This type of perseverative thinking is less abstract than the global self-criticism typical of non-traumatic depression and powerfully predicts chronicity (17, 18). Consequently, trauma-anchored depression tends to carry heavier shame, more comorbid anxiety, and a poorer response to standard SSRIs than depressions precipitated by an ongoing but time-limited stressor such as academic failure or family conflict (14, 19).

### Hypothesised mechanisms by which the regimen may have contributed to symptom reduction

#### Targeting rumination and intrusive threat loops

Dextromethorphan (DXM) delivers a brief, ketamine-like NMDA receptor blockade that, in theory, disrupts rigid cortico-striato-thalamo-cortical firing, a circuit implicated in both obsessive rumination and compulsive behaviour (20). Fluoxetine not only provides serotonergic support but, as a potent CYP2D6 inhibitor, may slow hepatic clearance of DXM so that this antiglutamatergic “reset” lasts for hours instead of minutes (21).

#### Re-building flexible synapses

Transient NMDA blockade triggers a glutamate “surge” at AMPA receptors; up-regulated AMPA throughput initiates BDNF- and mTOR-dependent synaptic remodelling that underlies the rapid lift in both mood and intrusive cognitions (22, 23). Piracetam, which binds to AMPA receptor dimer interfaces consistent with weak positive allosteric modulation (11) and has been reported to rescue extinction deficits and restore cortical and hippocampal physiology in a pre-clinical PTSD model (24), might in theory facilitate AMPA-mediated postsynaptic throughput, potentially strengthening the neuroplastic cascade and supporting longer-term cognitive stamina.

#### Practical advantages for an early adolescent

All three agents are orally available, inexpensive, and familiar to primary-care pharmacists, avoiding the cost and logistics of intravenous ketamine or off-label memantine (25). The regimen delivered a clinically significant PHQ-9 drop (23 → 15 in twelve days); no episodes of dissociation, hypertension, or cognitive dulling were clinically documented, though structured monitoring for these events was not performed—outcomes that satisfy the family’s wish for a “function-preserving” treatment.

Taken together, the trio directly addresses the hypothesised neurobiological signature of trauma-anchored depression—glutamate-driven rumination intertwined with fear circuitry—while remaining feasible for community practice. Continued monitoring will clarify whether booster cycles or dose adjustments are required as academic and social demands evolve. Generalisation of safety or efficacy to adolescents cannot be made from a single case; larger studies are required.

## Case 4: symptom changes in a patient with complex post-traumatic stress features, bipolar II, ADHD and borderline traits using an oral glutamatergic combination

### Case presentation

A 26-year-old Chinese woman was first seen in our outpatient clinic in June 2022 for pervasive low mood, anergia and social withdrawal of one-year duration. She reported a history of severe childhood adversity: parental abandonment, placement in a girls’ home at age 13 and repeated physical bullying throughout secondary school. Since late adolescence she had exhibited chronic hyperarousal, nightmares, intrusive memories of school assaults and an enduring sense of threat—features compatible with complex post-traumatic stress disorder (C-PTSD). Comorbid conditions had accumulated over time, including bipolar affective disorder (type II by history of hypomanic spending sprees and decreased need for sleep), attention-deficit/hyperactivity disorder (ADHD) and borderline personality traits with episodic self-harm by overdosing on antihistamines and hypnotics. There was no substance misuse and no major medical illness.

Initial mental-state examination revealed psychomotor retardation, restricted affect, passive suicidal ideation without plan and pronounced interpersonal sensitivity. Baseline self-report scores were in the severe range (PHQ-9 = 20, GAD-7 = 20). Numerous psychotropics had been tried before presentation, among them vortioxetine, sertraline, quetiapine, pregabalin, hypnotic z-drugs and low-dose benzodiazepines, each discontinued for lack of efficacy, sedation or weight gain. Stimulant treatment with lisdexamfetamine provided transient cognitive benefit but aggravated lability and insomnia. The picture was one of treatment-resistant depression on a background of complex trauma and neurodevelopmental vulnerability.

The fourth patient met ICD-11 criteria for Complex PTSD stemming from prolonged childhood adversity that encompassed parental abandonment, institutional placement, and repeated physical bullying. In addition to the standard PTSD triad—intrusive memories and nightmares of school assaults, habitual avoidance of social contact, and a chronic state of hyper-arousal—she exhibited the defining disturbances in self-organisation: severe affect dysregulation with mood lability and episodic self-harm, a persistently negative self-concept expressed as anergia and passive suicidal ideation, and enduring interpersonal instability with pronounced borderline traits. Comorbid conditions included Bipolar II Disorder with documented hypomanic episodes, Attention-Deficit/Hyperactivity Disorder, and treatment-resistant depression. Functional impairment was profound and long-standing. While DSM-5-TR lacks a formal Complex PTSD category, the presentation would be captured clinically as PTSD accompanied by Borderline Personality traits and Bipolar II Disorder.

### First glutamatergic strategy

In March 2025, after a further escalation in depressive and anxiety symptoms (PHQ-9 = 24, GAD-7 = 20) we initiated an oral combination of fluoxetine 20 mg every morning and dextromethorphan 30 mg at night (two 15 mg tablets) in an attempt to reproduce the mechanism of an approved dextromethorphan/bupropion formulation that was unavailable locally. Fluoxetine was selected deliberately for its potent CYP2D6 inhibition, expected to slow dextromethorphan metabolism and prolong central N-methyl-D-aspartate (NMDA) receptor blockade. Concomitant methylphenidate 36 mg prolonged-release was maintained for ADHD; existing hypnotics were left unchanged.

Within four weeks the patient described “mental quietening” and a tapered intensity of trauma-related rumination. Objective improvement paralleled her narrative: by 10 June 2025 PHQ-9 had fallen to 16 and GAD-7 to 10. She resumed craft hobbies, engaged more reliably in part-time study and displayed less reactivity in interpersonal situations. Sleep remained fragmented but daytime functioning was markedly better and no dissociative or cardiovascular adverse effects were observed.

### Loss of response

From mid-July the antidepressant effect appeared to wane. She reported avolition, binge spending and nocturnal sleep-walking; PHQ-9 climbed back to 23 on 19 August 2025. We raised the dextromethorphan dosage to 90 mg daily (three 15 mg tablets twice per day) and reduced fluoxetine to 10 mg to mitigate mild gastrointestinal discomfort, but mood benefits were minimal. The relapse coincided with renewed interpersonal conflicts, suggesting both neurobiological tolerance and psychosocial destabilisation.

### Augmentation with piracetam

On 16 September 2025 piracetam 600 mg twice daily was introduced, targeting post-synaptic α-amino-3-hydroxy-5-methyl-4-isoxazole-propionic acid (AMPA) receptor throughput to reinforce the plasticity cascade initiated by NMDA antagonism. All other psychotropics were kept unchanged. Two weeks later the patient spontaneously reported “clearer thinking” and an ability to awaken without excess methylphenidate. By 20 October 2025 mood and anxiety ratings had improved to PHQ-9 = 18, GAD-7 = 15. Attendance at vocational classes stabilised, impulse-driven online shopping subsided and no further episodes of self-harm occurred. She denied hallucinosis, mania or cognitive dulling; liver and renal profiles remained normal.

### Rationale for extending the “trio” to complex PTSD with bipolar, ADHD and borderline traits

It is hypothesised that the partial but rapid stabilisation observed after fluoxetine + dextromethorphan (DXM) may reflect engagement of the neurobiological “bottleneck” common to treatment-resistant trauma syndromes: depleted glutamate pools, down-regulated AMPA throughput, and loss of BDNF-driven spine density (1, 2). Complex PTSD magnifies that bottleneck by layering affect-switching (bipolar II), attentional noise (ADHD), and limbic hyper-reactivity (borderline personality disorder) onto an already eroded cortico-limbic network. Each comorbidity adds its own stress load and further suppresses synaptic plasticity, making conventional monoaminergic strategies too weak or too slow.

The three-part oral stack—DXM, a potent CYP2D6 inhibitor (fluoxetine in our case) and piracetam—is hypothesised to engage multiple stages of the plasticity cascade:

Initiation (NMDA blockade). DXM is an uncompetitive NMDA antagonist; when its clearance is slowed by fluoxetine it may produce a ketamine-like glutamate “burst” and open a transient learning window (2, 5).

Throughput (AMPA gain). Piracetam binds to multiple sites along the AMPA receptor dimer interface (11) and, in pre-clinical PTSD models, has been shown to rescue extinction deficits and restore physiological alterations in cortex and hippocampus (24); it is therefore hypothesised to contribute to AMPA-mediated throughput and the receptor bias thought necessary for BDNF release and fear-memory rewiring.

Downstream growth (BDNF-mTOR). The hypothesised net effect would be activation of mTOR signalling and spine formation—a mechanism analogous to that proposed for ketamine in chronic PTSD (3, 4).

For patients with bipolar diathesis, the regimen is appealing because it relies on glutamatergic—not dopaminergic—arousal and therefore carries less risk of mania than stimulant or dopamine-agonist augmentation. ADHD symptoms may also improve, as piracetam enhances prefrontal synchrony and DXM indirectly boosts catecholamine tone at sub-psychedelic doses (26). Borderline affect storms, which correlate with amygdala-hippocampal hyper-connectivity, could subside as the stack restores frontal inhibitory control and normalises extinction circuits (10).

A proof-of-concept case has already shown that DXM–bupropion (without piracetam or glutamine) rapidly reduced suicidality in a patient with depression, PTSD and borderline features (27). Our patient’s early gains on the simpler DXM + fluoxetine backbone, and her subsequent relapse, mirror that report and point to the same limitation: NMDA blockade alone may not hold the new synapses in place. Adding piracetam and glutamine aims to “lock in” the reconsolidated networks and could help sustain symptom improvement beyond the few-week plateau typical of ketamine monotherapy.

## Summary

### Key clinical outcomes and mechanistic insights

In this four-person case series (**Table 1**), temporal associations were noted between the introduction of an oral glutamatergic combination centred on fluoxetine-enhanced dextromethorphan (with optional piracetam and/or bupropion) and reported improvements in symptoms and functioning in four patients with trauma-spectrum presentations.

**Table 1:** Summary of the four cases.

	Case 1	Case 2	Case 3	Case 4
Patient Demographics	26-year-old Chinese male	28-year-old female nurse	13-year-old female student	26-year-old Chinese female
Primary Diagnosis & Trauma	Somatic PTSD (single undisclosed traumatic event)	Bereavement-related PTSD (mother’s suicide) on background Bipolar II Disorder	Trauma-linked major depressive episode (childhood bullying/betrayal)	Complex PTSD (childhood abandonment, bullying) with Bipolar II, ADHD, borderline traits
Key Baseline Symptoms	Daily flashbacks, severe diffuse bodily pain, panic attacks, hypervigilance, unemployment for 2 years. Baseline: PHQ-9 not reported, GAD-7 not reported	Intrusive images, nightmares, survivor guilt, hypersomnia, emotional numbing, occupational decline. Baseline: PHQ-9 27, GAD-7 21	Intrusive rumination, perfectionism, guilt, nocturnal worry, reduced enjoyment. Baseline: PHQ-9 23, GAD-7 21	Chronic hyperarousal, nightmares, intrusive memories, low mood, self-harm, interpersonal sensitivity. Baseline: PHQ-9 20–24, GAD-7 20
Regimen	DXM 45 mg/day + fluoxetine 20 mg/day (background: risperidone 0.5 mg, low-dose quetiapine, pregabalin, hypnotics)	DXM 60 mg/day + bupropion XL 150 mg/day + piracetam 1,200 mg/day (background: lamotrigine 100 mg, low-dose aripiprazole)	DXM 30 mg nightly + piracetam 600 mg nightly + fluoxetine 10 mg morning (plus risperidone 0.25 mg)	DXM 30–90 mg/day + fluoxetine 20 mg → 10 mg + piracetam 1,200 mg/day (background: methylphenidate 36 mg, hypnotics)
Time to Initial Improvement	2–8 weeks (pain/flashbacks markedly reduced by August 2024)	3 weeks (brighter affect, resolved hypersomnia, reduced nightmares/flashbacks)	12 days (“less stuck” on thoughts, easier sleep, regained pleasure in choir)	4 weeks initial (mental quietening); further gain 2 weeks after piracetam
Key Outcomes	Pain minimal → absent; flashbacks ceased; returned to full-time accounting job; PHQ-9 3 → 0–1, GAD-7 2 → 0–1; resumed leisure activities	Nightmares weekly → rare; returned to full-time nursing; PHQ-9 27 → 4, GAD-7 21 → 0; planning postgraduate study	Rumination eased; “brain quieter”; PHQ-9 23 → 15, GAD-7 21 → 13 (moderate range)	Reduced rumination, resumed hobbies/study; no further self-harm; PHQ-9 24 → 18, GAD-7 20 → 15
Follow-up Duration	14 months of sustained improvement	3+ months of sustained improvement (occasional intrusive thoughts remain)	Early follow-up only (regimen continued)	Ongoing (~7 months; partial, fluctuating improvement)
Safety Observations	No dissociation, hypertension, serotonergic toxicity, or mania clinically documented; structured screening for hypomania and serotonin syndrome was not performed	Mild tremor (responded to propranolol); no manic switch, hypertension, or psychosis clinically documented; structured screening for hypomania was not performed	No dizziness, dissociation, or GI upset reported by patient or parent; structured monitoring for these events was not performed	No dissociation, mania, or cognitive dulling clinically documented; structured screening was not performed. Liver and renal profiles remained normal

DXM, Dextromethorphan; PHQ-9, Patient Health Questionnaire-9; GAD-7, Generalized Anxiety Disorder-7; GI, Gastrointestinal. All outcomes represent temporal associations observed during routine clinical care. The term “improvement” denotes reductions in symptom-rating scores and subjective patient reports, not formal remission as defined by validated PTSD instruments (e.g., CAPS-5). Alternative explanations for the observed changes—including natural recovery, regression to the mean, expectancy effects, and contributions from concurrent medications or psychosocial support—cannot be excluded. Safety monitoring relied on clinical interview, spontaneous patient reports, and vital signs (including blood pressure); structured scales for hypomania/mania, serotonergic toxicity, or systematic organ-function panels were not routinely employed.

These observations are hypothesis-generating:

- Marked reductions in intrusive memories, ruminations, and somatic pain, usually within one to four weeks.
- Return to work or school after long periods of impairment.
- No dissociative, psychotic, or manic adverse events were clinically documented during follow-up, even in two patients with bipolar vulnerability; however, structured screening for hypomania/mania and serotonergic toxicity was not performed.
- Benefits that held steady for months without raising the dose.

**Figure 1:**
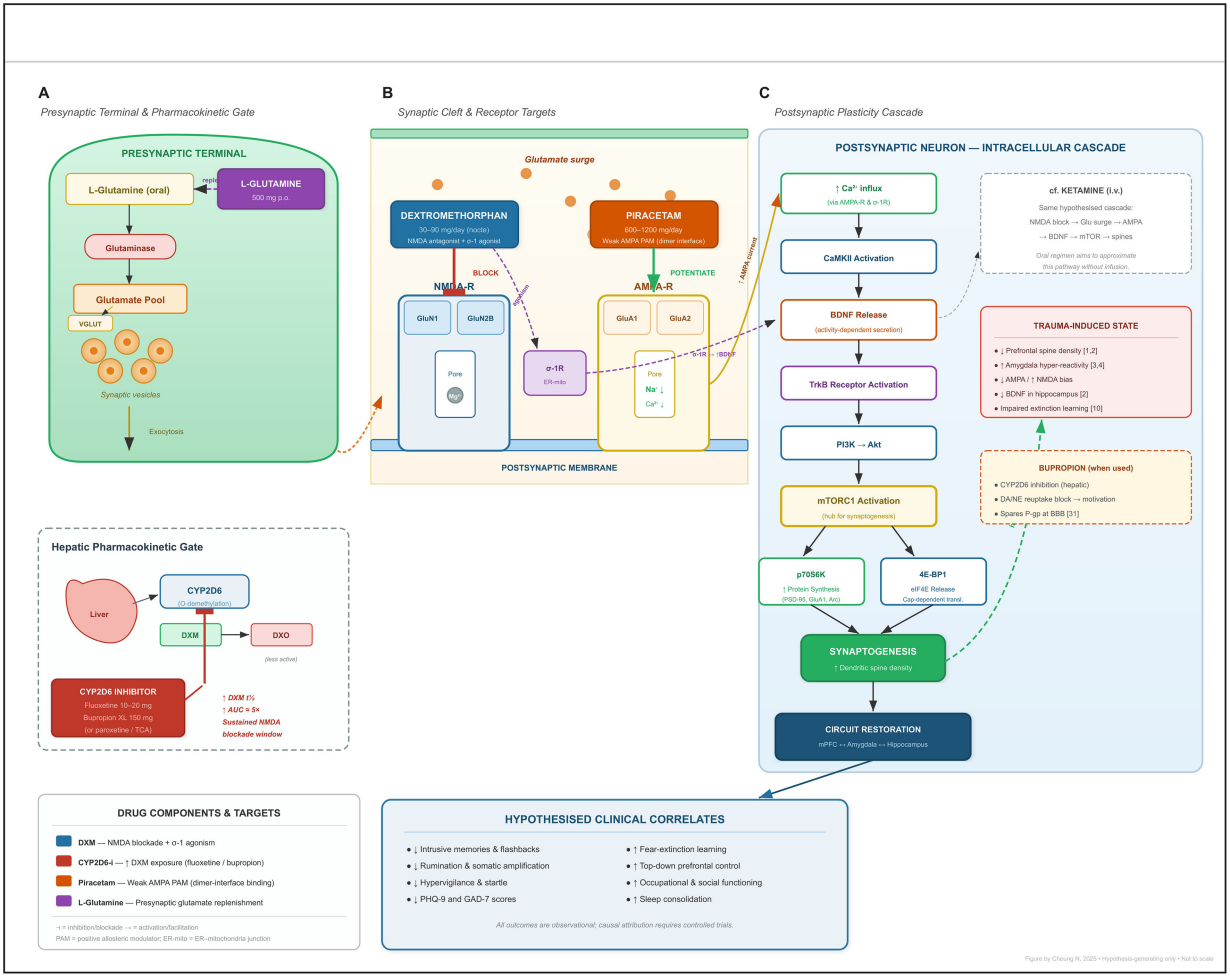
Hypothesised mechanistic model of the oral glutamatergic regimen for trauma-related disorders. The figure depicts the proposed pharmacological rationale for the four-component oral regimen and the intracellular plasticity cascade it is hypothesised to engage. **(A)** presynaptic terminal and pharmacokinetic gate. Oral L-glutamine is converted by glutaminase to glutamate, replenishing the presynaptic vesicular pool via vesicular glutamate transporters (VGLUT). In the hepatic inset, dextromethorphan (DXM) is normally O-demethylated to the less active metabolite dextrorphan (DXO) by cytochrome P450 2D6 (CYP2D6). A co-administered CYP2D6 inhibitor—fluoxetine, bupropion, or an alternative (red box)—blocks this conversion, raising systemic DXM exposure approximately five-fold and prolonging the therapeutic window of central NMDA receptor antagonism. **(B)** synaptic cleft and receptor targets. DXM exerts uncompetitive blockade at the GluN2B-containing NMDA receptor channel pore, reducing tonic NMDA-mediated signalling and precipitating a compensatory glutamate “surge” into the cleft. DXM simultaneously acts as a σ-1 receptor agonist at the endoplasmic reticulum–mitochondria interface, which is proposed to enhance BDNF secretion via a parallel route. Piracetam, a weak positive allosteric modulator that binds at the AMPA receptor dimer interface (GluA1/GluA2 subunits) (11), is hypothesised to potentiate the AMPA-mediated component of the glutamate surge, biasing postsynaptic throughput toward the rapid, depolarising current required for downstream plasticity signalling. **(C)** postsynaptic plasticity cascade. Enhanced AMPA-mediated Ca²^+^ influx activates calcium/calmodulin-dependent protein kinase II (CaMKII), triggering activity-dependent BDNF release. BDNF engages tropomyosin receptor kinase B (TrkB), which signals through PI3K–Akt to activate the mechanistic target of rapamycin complex 1 (mTORC1). mTORC1 in turn phosphorylates p70S6 kinase and eIF4E-binding protein 1 (4E-BP1), initiating cap-dependent translation of synaptic proteins including PSD-95, GluA1, and Arc. The net result is hypothesised to be increased dendritic spine density (synaptogenesis) in prefrontal–hippocampal–amygdalar circuits, supporting restoration of top-down inhibitory control and fear-extinction capacity. This cascade parallels the mechanism attributed to intravenous ketamine (dashed comparator box) but is proposed to be achievable with inexpensive, orally administered agents taken in routine outpatient settings. The red annotation box lists the synaptic deficits induced by chronic trauma that the regimen is hypothesised to reverse (1–4, 10). All mechanistic attributions in this figure are hypothesis-generating; causal demonstration requires controlled trials with neuroimaging and validated trauma-specific outcome measures. Bracketed numbers refer to manuscript references. AMPA-R, α-amino-3-hydroxy-5-methyl-4-isoxazolepropionic acid receptor; BBB, blood–brain barrier; BDNF, brain-derived neurotrophic factor; CaMKII, calcium/calmodulin-dependent protein kinase II; DA, dopamine; DXM, dextromethorphan; DXO, dextrorphan; eIF4E, eukaryotic initiation factor 4E; ER-mito, endoplasmic reticulum–mitochondria junction; mTORC1, mechanistic target of rapamycin complex 1; NE, norepinephrine; NMDA-R, N-methyl-D-aspartate receptor; PAM, positive allosteric modulator; P-gp, P-glycoprotein; PI3K, phosphoinositide 3-kinase; PSD-95, postsynaptic density protein 95; σ-1R, sigma-1 receptor; TrkB, tropomyosin receptor kinase B; VGLUT, vesicular glutamate transporter; 4E-BP1, eIF4E-binding protein 1.

Biologically, the combination is hypothesised to engage mechanisms similar to those proposed for ketamine: removing the NMDA “brake,” boosting AMPA signalling, and driving a BDNF–mTOR surge that might contribute to disrupting fear-based circuits and restoring top-down control (**Figure 1**). Fluoxetine (or bupropion) prolongs dextromethorphan’s action; piracetam may facilitate postsynaptic AMPA throughput (11); together they are hypothesised to approximate aspects of ketamine-facilitated plasticity in a daily, oral format.

The favourable outcomes seen across these four cases may also be considered in the context of a recently proposed hypothesis linking PTSD vulnerability to developmental synaptic pruning. In a preprint by the present author (28), it was suggested that common alleles regulating microglial complement activity (C4A), MHC-I expression (HLA-B), and axon-guidance cues such as SEMA3F and EFNA5 may bias the adolescent brain toward excessive synaptic pruning. If confirmed by independent groups, corticolimbic networks would consequently be left under-refined; when a major stressor later drives a surge in glutamatergic transmission, those immature circuits may fail to stabilise, fear-extinction learning may collapse, and full-blown PTSD may emerge. From this speculative perspective, the clinical responses observed with the oral NMDA–AMPA regimen may represent a hypothesised partial reversal of the second phase of the cascade—”trauma-triggered plasticity failure”—by re-establishing synaptic density and connectivity in pruning-sensitive regions.

Preliminary observations from the same author’s clinical practice, published as preprints, have described similar patterns of symptom change using comparable glutamatergic combinations. A middle-aged patient with chronic derealisation and fugue-like episodes following childhood adversity experienced cessation of fugue spells and substantial relief of derealisation within six weeks of starting fluoxetine-potentiated dextromethorphan 30 mg plus piracetam titrated to 1–200 mg daily; full occupational functioning returned soon afterwards (29). In another case, a young woman with complex PTSD related to prolonged school bullying attained partial improvement over four months with a stack of bupropion-potentiated dextromethorphan (up to 60 mg), piracetam 1–200 mg, and L-glutamine, reporting disappearance of somatic anxiety and a pronounced drop in ruminative thinking (30). Additional single-case observations describe rapid dampening of flashbacks, nightmares, and suicidal ideation in trauma-linked presentations, again using oral NMDA antagonism combined with AMPA facilitation (31). These reports are uncontrolled observations from a single clinician’s practice and should be interpreted with that limitation in mind; they do not constitute independent replication.

Taken together, these converging but preliminary observations suggest that inexpensive, orally administered modulators of NMDA and AMPA receptors can be administered in everyday practice without clinically documented serious adverse events in these cases, and may target the synaptic destabilisation hypothesised to underlie trauma-related disorders. While controlled trials remain essential, the mechanistic bridge between genetic vulnerability, stress-induced synaptic collapse, and drug-facilitated plasticity offers a hypothesis-generating framework for further exploration.

## Limitations, comorbidities, and polypharmacy considerations

The work is limited by its retrospective nature, small sample, concurrent medications, lack of structured PTSD ratings (e.g., CAPS-5), and the inability to tease apart each drug’s contribution. Alternative explanations for the observed improvements, including natural recovery, regression to the mean, expectancy effects, and contributions from concurrent medications or psychosocial support, cannot be excluded.

A final issue that deserves attention is the tangle of comorbidity and polypharmacy that characterised every patient in this series. Bipolar II disorder, ADHD, borderline traits, and longstanding prescriptions for mood stabilisers or antipsychotics were the rule rather than the exception. Such clinical complexity mirrors everyday practice in treatment-resistant, trauma-spectrum illness, but it makes it difficult to assign the observed gains solely to the glutamatergic stack. It is entirely plausible that the co-administered agents added something useful—dopaminergic tone from stimulants, GABAergic buffering from benzodiazepines, or mood-steadying effects from lithium—that helped keep the “plasticity window” open. By the same token, each additional drug raises the spectre of kinetic or dynamic interactions that could blunt efficacy or magnify risk.

Nowhere is this balancing act clearer than in the choice of the CYP2D6 inhibitor used to boost dextromethorphan. Paroxetine, with its irreversible “suicide” inhibition, and fluoxetine, with a long-lived norfluoxetine metabolite, can drive plasma levels of other 2D6 substrates—risperidone, aripiprazole, many tricyclics—into a range associated with serotonin toxicity, akathisia, or mood destabilisation. Bupropion offers potent 2D6 blockade while sparing P-glycoprotein at the blood–brain barrier, an advantage when stimulant exposure is desirable. Duloxetine provides a moderate, quickly reversible effect that suits patients who may need rapid dose adjustments, whereas very-low-dose tricyclics furnish the gentlest option when the risk of a manic switch looms large (32). Selecting the inhibitor therefore becomes a bespoke decision, dictated by the existing medication list and the patient’s affective volatility.

Similar dilemmas arise when obsessive–compulsive symptom clusters overlap with trauma, a scenario that often prompts additional serotonergic or antipsychotic prescriptions. The resulting polypharmacy muddies causal inference yet reflects the real-world setting in which clinicians rarely withdraw anchor drugs simply to test a new intervention (33). In such cases, slow titration, conservative target doses, and close surveillance for emergent agitation, hypertension, or hepatic strain appear pivotal. Taken together, the cases underline a pragmatic lesson: the promise of oral NMDA–AMPA modulation can be realised in multiply medicated patients, but only when inhibitor selection is thoughtful and vigilance for drug–drug interaction is built into routine follow-up.

### Implications for future research

These early results support the rationale for prospective trials of oral NMDA–AMPA modulators as an affordable area for investigation between ketamine infusion clinics and routine community care. Randomised studies with neuroimaging, trauma-specific outcome measures, and direct comparisons with Auvelity are now needed.

## Data Availability

The original contributions presented in the study are included in the article/supplementary material. Further inquiries can be directed to the corresponding author.
